# Targeted Sequencing Analysis of Matched Cell-Free DNA and White Blood Cells: A Facile Method for Detection of Residual Disease in Gastric Cancer

**DOI:** 10.1055/s-0040-1716333

**Published:** 2020-08-31

**Authors:** Feiyu Diao

**Affiliations:** 1 Department of General Surgery, Sun Yat-Sen Memorial Hospital, Sun Yat-Sen University, Guangzhou, People's Republic of China

Gastric cancer (GC) is one of the most diagnosed cancers in the world and is mainly cured by surgical resection. However, a large proportion of patients still presents with microscopic residual disease after surgical resection. Patients with microscopic residual disease can be offered appropriate treatment to improve their long-term survival, but for such, it is imperative to timely identify those with residual disease after surgery. Unfortunately, the traditional detection methods for residual disease have poor sensitivity. Recently, with the development of liquid biopsy technology, circulating tumor-derived deoxyribonucleic acid (ctDNA) detection has shown great promise in bringing new hope for increasing the detection rate of microscopic residual disease. In a study recently published in *Nature Communications* , Leal et al developed a novel liquid biopsy strategy to distinguish tumor-specific ctDNA alterations from cell-free DNA variants associated with clonal hematopoiesis and evaluated the utility of ctDNA as a biomarker for predicting the prognosis of postoperative GC patients.

Gastric cancer (GC) is the fifth common cancer and third leading cause of cancer-related death worldwide. 1 Although the incidence of GC has declined rapidly over recent decades; however, compared with other countries, this decline has been less striking in China. 2 Additionally, the prognosis of GC patients remains poor in China. 3 The curative treatment for GC is based on R0 resection with D2 lymphadenectomy. 4 However, a large proportion of patients still presents with microscopic residual disease after surgery and eventually succumb due to local recurrence or distant metastases. 5 Patients with microscopic residual disease can be offered appropriate treatment to improve their long-term survival if they are timely identified. 4 Unfortunately, the currently available detection methods for residual diseases, such as traditional blood biomarker detection and imaging techniques, have poor sensitivity, specificity, and cannot be reliably used. 6

With the development of liquid biopsy technology, the latest approaches, such as circulating tumor-derived deoxyribonucleic acid (ctDNA) detection via liquid biopsy, may bring new hope for detecting microscopic residual disease after GC surgery. Theoretically, the tumor-specific alterations in the circulation of postoperative cancer patients can dynamically and specifically reflect whether these patients have microscopic residual disease or preclinical metastases. 7 8 Recently, ctDNA released from cancer cells into the peripheral blood has been noninvasively detected, not only in late-stage cancer patients but also in those at early stage. 9 The most critical step of liquid biopsy is to distinguish tumor-specific ctDNA from large amounts of cell-free DNA. Most of the previous studies only focused on alterations in ctDNA during anticancer treatment and mainly on the occurrence of metastatic disease. All of them have only analyzed a limited number of genomic positions that only represent a small subset of tumor clones. 10 11 More recent studies have started to apply blood-based deep sequencing approaches to detect white blood cell-derived variants that associate with clonal hematopoiesis in cell-free DNA. 9 12 Additionally, such studies also used similar approaches to evaluate the white blood cell DNA and cell-free DNA from cancer patients at a single time point. 9 13 However, no study has yet attempted to evaluate these during anticancer treatment to predict prognosis. In a study recently published in *Nature Communications* , titled as “White blood cell and cell-free DNA analyses for detection of residual disease in gastric cancer,” Leal et al 14 developed a novel liquid biopsy strategy to distinguish tumor-specific ctDNA alterations from cell-free DNA variants associated with clonal hematopoiesis and evaluated the utility of ctDNA as a biomarker for predicting the prognosis of postoperative GC patients.

All GC patients enrolled in that study were from the CRITICS trial (NCT00407186). 15 The plasma from each patient was collected at the time of trial enrollment (baseline), preoperatively, and after surgery but before postoperative chemotherapy (median time after surgery = 6.5 weeks). The novel approach developed in this study was as follows: the cell-free DNA and white blood cells in blood samples were first analyzed by parallel depth sequencing, and then the tumor-specific alterations in blood samples were identified by removing the hematopoietic-associated alterations detected in white blood cells from the cell-free DNA data. The blood samples from 50 GC patients were evaluated using this new approach. Among them, 27 patients harbored tumor-specific alterations at baseline. In addition, the detection of ctDNA variants at baseline did not show statistically significant differences in both event-free and overall survival. Next, the authors evaluated ctDNA levels in the blood samples collected at the baseline and after preoperative chemotherapy. They found that the preoperative ctDNA level was positively correlated with pathological response in GC patients. Finally, the authors evaluated microscopic residual disease in all 20 patients who had blood samples collected after surgery. They found that tumor-specific alterations in cell-free DNA from 4 patients with major tumor responses disappeared completely after surgery. However, postoperative tumor-specific alterations were detectable in 9 out of 16 patients with minor tumor responses. All 11 patients without detectable tumor-specific alterations after surgery were free of recurrence, while 6 out of 9 patients with detectable tumor-specific alterations after surgery developed metastatic disease.

Furthermore, the detection of microscopic residual disease without a white blood cell filter did not predict disease recurrence. In contrast, with a white blood cell filter, a significantly shorter median event-free survival, and a significantly higher risk of disease recurrence for patients with detectable tumor-specific mutations after surgery could be observed. All results mentioned above suggested that microscopic residual disease could be accurately detected by the new detection method developed in the study by Leal et al, and microscopic residual disease could be a predictive indicator for the prognosis of postoperative GC patients.

Patients with microscopic residual disease would need adjuvant treatment after surgery. It is important to diagnose them as soon as possible. At present, we still rely on traditional methods, such as pathological staging and microscopic residual disease scoring system, to estimate the risk of GC recurrence after surgery. 16 However, these methods have some limitations, which prevent them from being used in clinical practice. 16 Furthermore, the sensitivity of currently available imaging and blood biomarker detection methods is poor. 6 The above-discussed study is the first to develop a tissue-independent detection method that detects tumor-specific mutations in the cell-free DNA of GC patients before and after surgery by sequencing the matched white blood cell and cell-free DNA. In this study, the authors first investigated the value of white blood cell and cell-free DNA parallel deep sequencing to detect clonal hematopoiesis-associated cell-free DNA alterations. After that, they used this method to infer the bona fide alterations of tumor longitudinally. Although the detection of microscopic residual disease by analyzing ctDNA has been used in a variety of tumors, 7 10 the new strategy developed in this study still has many advantages. For example, this strategy can identify ctDNA without tumor tissue at any time point before and after surgery. In addition, its detection data will not be affected by the heterogeneity within the tumor mass. Even though this study only analyzed a small number of GC patients with sufficient plasma samples, the extensive follow-up time of this study was sufficient to accurately determine clinical recurrences. Moreover, although the panel used in this study was not developed for GC, at the baseline time point, the majority of the GC patients were still detected at the baseline time point. About 60% of GC who were not detected at baseline had a diffuse subtype morphology. This finding suggests that the histological features of GC may not be related to tumor-specific DNA shedding. Furthermore, some GC patients did not relapse although their ctDNA results detected by the new method were positive. The best explanation for this observation could be that these patients were cured after receiving adjuvant therapy, but Leal et al did not assess their ctDNA levels after receiving adjuvant therapy.

Overall, the discussed study developed a facile approach for detecting microscopic residual disease in GC by distinguishing ctDNA alterations from other cell-free DNA alterations. However, when using this new approach to detect postoperative microscopic residual disease of GC, there are some limitations, especially in GC patients who have localized diseases. Therefore, in the future, it is necessary to improve this approach by using targeted panels specifically designed for GC and incorporating cell-free DNA fragmentation analyses.
